# Soluble amyloid beta levels are elevated in the white matter of Alzheimer’s patients, independent of cortical plaque severity

**DOI:** 10.1186/s40478-014-0083-0

**Published:** 2014-08-17

**Authors:** Lyndsey E Collins-Praino, Yitshak I Francis, Erica Y Griffith, Anne F Wiegman, Jonathan Urbach, Arlene Lawton, Lawrence S Honig, Etty Cortes, Jean Paul G Vonsattel, Peter D Canoll, James E Goldman, Adam M Brickman

**Affiliations:** Taub Institute for Research on Alzheimer’s Disease and the Aging Brain, College of Physicians and Surgeons, Columbia University, 630 West 168th Street, New York, NY 10032 USA; Department of Anatomy and Pathology, University of Adelaide, Adelaide, South Australia Australia; Department of Neurology, College of Physicians and Surgeons, Columbia University, New York, NY USA; Department of Pathology and Cell Biology, College of Physicians and Surgeons, Columbia University, New York, NY USA

**Keywords:** Dementia, Neurodegenerative disease, Pathology, Myelin, Ageing, ELISA

## Abstract

Alzheimer’s disease (AD) is the most common neurodegenerative disease and the leading cause of dementia. In addition to grey matter pathology, white matter changes are now recognized as an important pathological feature in the emergence of the disease. Despite growing recognition of the importance of white matter abnormalities in the pathogenesis of AD, the causes of white matter degeneration are still unknown. While multiple studies propose Wallerian-like degeneration as the source of white matter change, others suggest that primary white matter pathology may be due, at least in part, to other mechanisms, including local effects of toxic Aβ peptides. In the current study, we investigated levels of soluble amyloid-beta (Aβ) in white matter of AD patients (n=12) compared with controls (n=10). Fresh frozen white matter samples were obtained from anterior (Brodmann area 9) and posterior (Brodmann area 1, 2 and 3) areas of post-mortem AD and control brains. ELISA was used to examine levels of soluble Aβ -42 and Aβ -40. Total cortical neuritic plaque severity rating was derived from individual ratings in the following areas of cortex: mid-frontal, superior temporal, pre-central, inferior parietal, hippocampus (CA1), subiculum, entorhinal cortex, transentorhinal cortex, inferior temporal, amygdala and basal forebrain. Compared with controls, AD samples had higher white matter levels of both soluble Aβ -42 and Aβ -40. While no regional white matter differences were found in Aβ -40, Aβ -42 levels were higher in anterior regions than in posterior regions across both groups. After statistically controlling for total cortical neuritic plaque severity, differences in both soluble Aβ -42 and Aβ -40 between the groups remained, suggesting that white matter Aβ peptides accumulate independent of overall grey matter fibrillar amyloid pathology and are not simply a reflection of overall amyloid burden. These results shed light on one potential mechanism through which white matter degeneration may occur in AD. Given that white matter degeneration may be an early marker of disease, preceding grey matter atrophy, understanding the mechanisms and risk factors that may lead to white matter loss could help to identify those at high risk and to intervene earlier in the pathogenic process.

## Introduction

Alzheimer’s disease (AD) is the most common neurodegenerative disease and the leading cause of dementia [1]. Pathologically, AD is characterized by neuronal loss, amyloid-beta (Aβ) plaques, composed of aggregations of amyloid peptides, and neurofibrillary tangles (NFT), consisting of hyperphosphorylated tau protein. The “amyloid cascade hypothesis”, currently the prevailing hypothesis regarding AD pathogenesis, states that Aβ-42 peptides aggregate to form toxic Aβ-42 oligomers and plaques, which then trigger a cascade of neuropathological events, including neuroinflammation, oxidative stress, tau hyperphosphorylation and NFT formation, and, ultimately, widespread neurodegeneration and dementia [2].

Although AD has traditionally been considered a disease of the grey matter, in recent years, white matter changes have come to be recognized as an important pathological feature [3,4]. Using structural magnetic resonance imaging (MRI), both white matter atrophy [5-8] and white matter lesions that appear as areas of white matter hyperintensity (WMH) on T2-weighted magnetic resonance imaging (MRI) sequences [9-13] have been reported in AD. At the microstructural level, diffusion tensor imaging (DTI) studies found reductions in white matter integrity in AD patients in multiple white matter regions [3,14-22]. In neuropathology studies, white matter abnormalities have been found to occur in more than 50% of AD cases [23], and include axonal defect and loss, demyelination, death of oligodendrocytes, reactive astrocytosis and microglial activation [23-33]. White matter abnormalities are associated with performance across a range of neuropsychological tests [34], and predict AD incidence and rate of cognitive decline in the community [9,35-37]. Although once thought to occur secondary to grey matter degeneration, recent studies demonstrated that some white matter changes in AD may occur independently of and precede grey matter atrophy [3,4].

Despite the growing recognition of the importance of white matter abnormalities in the pathogenesis of AD, the pathological basis of white matter degradation remains to be elucidated. While multiple studies attributed white matter degeneration to concomitant small vessel disease [23,32] or Wallerian-like degeneration [4,32], others suggest that primary white matter pathology may be due, at least in part, to other mechanisms, including toxic Aβ peptides [4]. To investigate this possibility further, we measured levels of soluble Aβ-40 and Aβ-42 peptides in postmortem cerebral white matter from AD patients and non-AD controls. We elected to measure soluble amyloid levels as, in recent years, extensive evidence has accumulated to suggest that it is the levels of soluble Aβ oligomers, rather than insoluble Aβ fibrils, that correlate with the extent of synaptic loss and the severity of cognitive impairment in AD [38-44]. Given the amyloid cascade hypothesis [2] and the known increase in Aβ in AD brains, we hypothesized that AD patients would show increased white matter levels of soluble Aβ-40 and Aβ-42 peptides compared with non-AD controls. We also hypothesized that white matter amyloid beta levels would still be increased in AD brains, even after statistically controlling for cortical plaque severity, suggesting that soluble forms of amyloid in the white matter of AD patients may not merely be a reflection of overall amyloid pathology, but rather could independently contribute to the pathophysiology of the disease. While neuropathological studies have shown that the frontal lobes appear to be the most severely affected by white matter abnormalities [23], we have found using structural MRI that frank white matter damage to more posterior areas is associated with incident AD [9,35]. Given this disparity between pathological and imaging findings, the current study also used tissue sections from both anterior and posterior sections to investigate whether white matter amyloid beta levels vary as a function of region.

## Materials and methods

### Participants and neuropathological assessment

Twenty-two cases from the New York Brain Bank at Columbia University were included in the current study (see Table 1 for demographic information). Twelve of these cases were pathologically diagnosed as AD and 10 were non-AD controls. Within the control group, 3 cases were pathologically normal, 4 were classified pathologically as ischemic stroke without vascular dementia, 1 was classified with progressive supranuclear palsy, 1 with idiopathic Parkinson’s disease and 1 with lobar atrophy without Picks disease. Neuropathological assessment was performed blind to clinical diagnosis and diagnosis of AD was based on CERAD criteria [45]. Of the twelve clinical AD patients, eight had definite AD and four had probable AD based upon CERAD criteria [46]. Of the ten non-Alzheimer’s controls, nine did not meet CERAD criteria for AD and one was classified as having a low probability of AD [46]. As these are historic cases, they have not been re-evaluated according to the more recent National Institute on Aging-Alzheimer’s Association (NIA-AA) criteria. In addition to CERAD criteria, however, diagnosis was confirmed by also rating each case according to the Braak and Braak neuropathological staging criteria for Alzheimer-related changes [47] and the recommendations of the National Institute on Aging and Reagan Institute (NIA-RI) [48]. Together, these three ratings are largely equivalent to the more contemporary NIA-AA criteria [49]. Within the Alzheimer’s group, the ApoE status of nine of the twelve cases was known; of these, four had an ε4 allele. The ApoE status of nine of the ten cases in the non-Alzheimer’s control group was known; of these, three had an ε4 allele.

**Table 1:** **Demographic data**

	**AD (n=12)**	**Non-AD controls (n=10)**
Age, years, mean (SD)	89.00 (10.43)	81.00 (7.70)
Male, %	25%	50%
Black, %	8.33%	20%
Hispanic, %	33.33%	30%

This research was reviewed by the Chair of the Columbia University Medical Center Institutional Review Board, who deemed the work exempt from further review (under 45 CFR 46) because there was no interaction with human subjects, no intervention, and private, identifiable information was not collected.

The tissue processing procedure has been described in detail previously [45]. Briefly, the whole fresh brain was first examined grossly, photographed and weighed. The brain was then divided into two halves. One half brain was processed fresh and frozen samples banked for research. The other half brain was formalin fixed processed for thorough neuropathological evaluation.

### White matter dissection

For the current study, fresh frozen tissue blocks from Brodmann area (BA) 1, 2, and 3 (primary somatosensory cortex, posterior sections) and BA9 (dorso-lateral prefrontal cortex, anterior sections) were utilized. White matter was manually dissected from each block on dry ice to prevent thawing of the tissue and frozen at −80°C until biochemical analysis.

### Analysis of amyloid levels

White matter samples (2 g each) were homogenized via sonication on ice in 880 μL of tissue lysate buffer (20 mM Tris–HCl (pH 7.4), 1 mM EDTA, 1 mM ethyleneglycoltetraacetic acid, 250 mM sucrose) supplemented with protease inhibitors (Roche). The tissue homogenates were treated with diethanolamine to extract soluble Aβ and centrifuged at 100,000 × g for 60 minutes at 4°C. The definition of soluble *versus* insoluble Aβ was the same as that used in previous studies [40,50]: that is, molecules that remain in the aqueous supernatant after centrifugation for 1 hour are considered soluble Aβ, while those Aβ aggregates that remain in the pellet are considered as insoluble Aβ. The supernatant was collected, total protein concentration was determined by a BCA protein assay (Thermo Scientific) and homogenate concentrations were standardized. Aβ-40 levels were determined using the Aβ-40 Type II ELISA kit from Wako (Catalog Number: 292–64701). Aβ-42 levels were measured using the Aβ-42 High Sensitivity ELISA kit from Wako (Catalog number: 292–64501). These kits have been extensively validated in previous studies (e.g. [51-55]) and are known to show extremely high sensitivity and reproducibility [54]. Both ELISA assays were performed in accordance with the manufacturer’s protocol, and all samples were run in duplicate. Optical density values were measured at 450 nm using a microplate reader, and then converted to concentrations (pmol/L) based on a standard curve. For cortical amyloid plaque ratings, a trained pathologist examined individual tissue sections and the number of Aβ plaques was manually counted. Neuritic plaque severity was rated in one section of each of the following areas from the fixed hemisphere: mid-frontal, superior temporal, pre-central, inferior parietal, hippocampus (CA1), subiculum, entorhinal cortex, transentorhinal cortex, inferior temporal, amygdala and basal forebrain. For each cortical area, a neuropathologist scanned the cortex over the entire slide, picked the most involved area, and then counted neuritic plaques stained with a Bielschowsky stain using the 10x ocular and 10x objective lenses. Each cortical region received a severity rating based on the following: 1, if there were less than 5 neuritic plaques, 2, if the number of neuritic plaques was between 5 and 15 and 3, if there were more than 15 neuritic plaques. We derived the total cortical neuritic plaque severity rating from the individual rating of each cortical region as follows: 1, if the total neuritic plaque rating was mild (i.e. the majority of cortical areas contained fewer than 5 Aβ plaques), 2, if the total neuritic plaque rating was moderate (i.e. the majority of cortical areas contained between 5 and 15 Aβ plaques) and 3, if the total neuritic plaque rating was severe (i.e. the majority of cortical areas contained more than 15 Aβ plaques). White matter tissue immunostained with Aβ antibodies from each case included in the study was also examined for the presence of white matter neuritic plaques, but none were found in any section.

### Data analysis

Data were first analyzed with a repeated measures analysis of variance (ANOVA), with Region (2 levels: Anterior, posterior) as a within-subjects variable and Group (2 levels: AD, control) as a between-subjects variable. Two separate ANOVAs were conducted for Aβ-40 levels and Aβ-42 levels. Age at death was included as a covariate. In order to investigate further the effect of region, the nonparametric Wilcoxon signed rank sum test was used. To study the relationship between white matter Aβ levels and cortical plaque burden, Pearson correlations were calculated between white matter Aβ levels and neuritic plaque severity rating in several cortical areas. To investigate whether white matter levels of soluble Aβ were different between AD cases and non-AD controls, independent of cortical plaque pathology, a measure of total cortical neuritic plaque severity was included as a covariate and each ANOVA was re-run to test if the effect of either Group (AD vs. control) or Region (anterior vs. posterior) was still statistically significant.

## Results

### *A*β*-40 levels*

**Figure 1:**
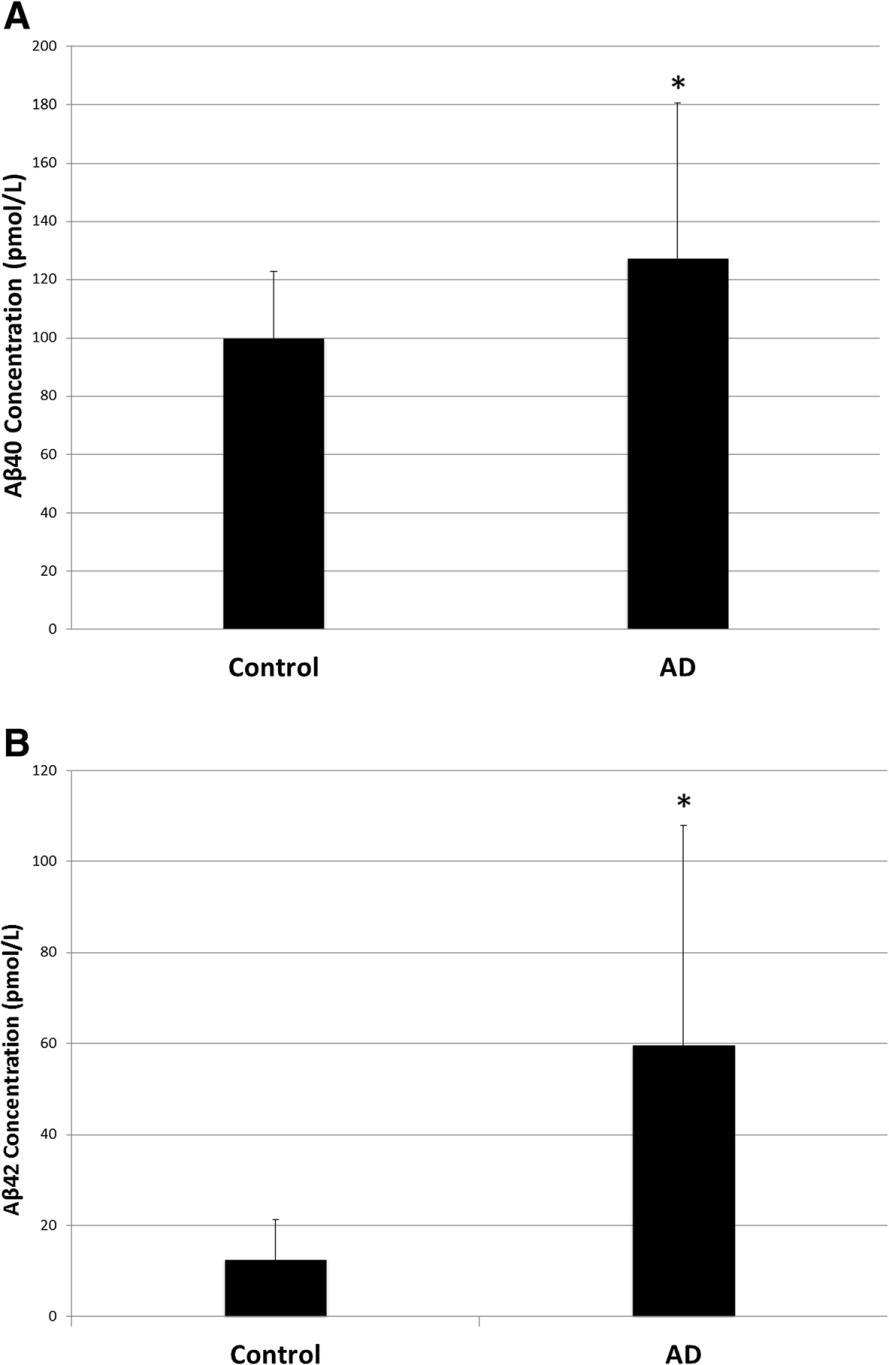
**Main effect of group. (A)** Average Aβ-40 levels were higher in the white matter of AD patients compared with non-AD controls. **(B)** Aβ-42 levels were significantly higher in the white matter of AD patients compared with non-AD controls.

**Figure 2:**
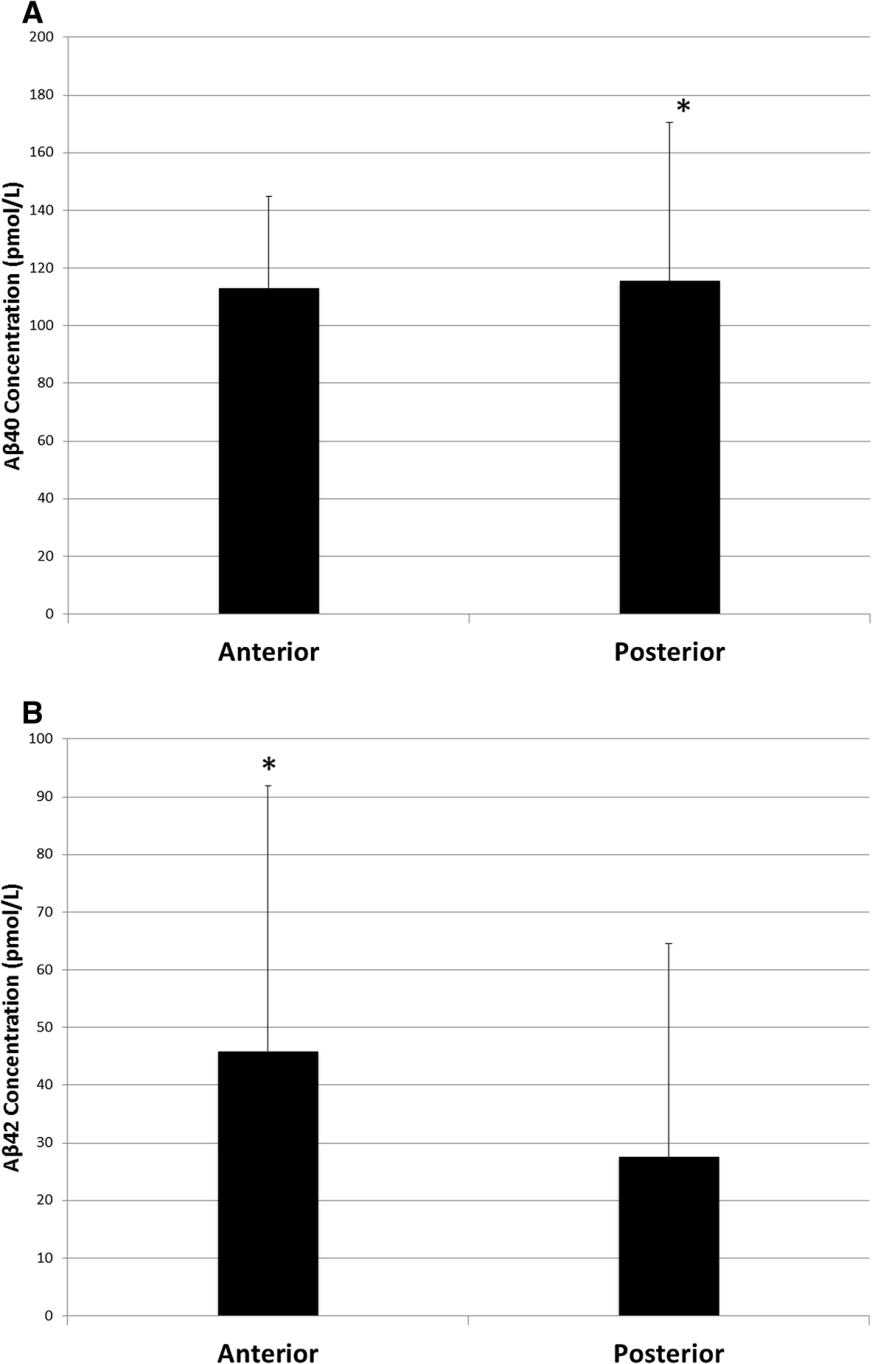
**Main effect of region. (A)** Aβ-40 levels were not significantly different in anterior or posterior regions of white matter. **(B)** There was a trend for Aβ-42 levels to be increased in anterior regions of white matter compared to posterior regions.

AD patients had higher average white matter Aβ-40 levels than non-AD controls (main effect of Group: F(1,15) = 6.338, *p* = 0.024; Figure 1A). Aβ-40 levels did not differ between anterior and posterior white matter regions in either the overall ANOVA (main effect of Region: F(1,15) = 0.718, *p* = 0.410; Figure 2A) or when the regional effect was further probed using a Wilcoxon signed-ranks test (Z = −0.675, *p =* 0.500). There was not an interaction between group and region (Group x Region interaction: F(1,15) = 0.003, *p* = 0.957). No significant correlations were found between either anterior or posterior white matter levels of Aβ-40 and neuritic plaque severity rating in any cortical region (Table 2). After statistically controlling for total cortical neuritic plaque severity, Alzheimer’s patients still had higher average white matter Aβ-40 levels than non-Alzheimer’s controls (main effect of Group: F(1,14) = 6.312, *p* = 0.025). After statistically controlling for total cortical neuritic plaque severity, there was still neither a main effect of region (F(1,14) = 0.940, *p =* 0.349) nor an interaction between group and region (Group x Region interaction: F(1,14) = 0.141, *p* = 0.713).

**Table 2:** **Correlations between white matter Aβ 40/42 levels (Anterior and posterior) and plaque severity rating, assayed by CERAD criteria, in several CNS regions**

	**Ant. Aβ-40**		**Post. Aβ-40**		**Ant. Aβ-42**		**Post. Aβ-42**	
	**r**	**p**	**r**	**p**	**r**	**p**	**r**	**p**
Mid-frontal	0.294	0.196	0.204	0.403	**0.697**	**<0.001**	**0.630**	**0.004**
Superior temporal	-0.263	0.529	0.194	0.617	0.607	0.111	**0.706**	**0.034**
Pre-central	0.042	0.862	0.244	0.329	**0.497**	**0.026**	0.413	0.087
Inferior Parietal	0.351	0.118	0.228	0.347	**0.744**	**<0.001**	**0.633**	**0.004**
Hipp-CA1	0.019	0.937	0.082	0.746	**0.618**	**0.004**	**0.596**	**0.009**
Subiculum	0.282	0.228	0.347	0.159	**0.457**	**0.043**	0.448	0.062
Entorhinal	0.227	0.336	0.348	0.158	**0.478**	**0.033**	**0.498**	**0.035**
Transentorhinal	0.220	0.352	0.349	0.155	**0.565**	**0.009**	**0.584**	**0.011**
Inferior Temporal	0.270	0.250	0.298	0.230	**0.653**	**0.002**	**0.655**	**0.003**
Amygdala	0.365	0.103	0.132	0.591	**0.618**	**0.003**	**0.482**	**0.037**
Basal forebrain	0.360	0.109	-.024	0.922	**0.442**	**0.045**	0.169	0.488

Note: Significant correlations are indicated by bolded text.

### *A*β*-42 levels*

Compared with non-AD controls, AD patients had higher average levels of Aβ-42 (main effect of Group: F(1,15) = 8.024, *p* = 0.013; Figure 1B). While Aβ-42 levels were not different in anterior and posterior white matter regions (main effect of Region: F(1,15) = 1.001, *p* = 0.333; Figure 2B), there was a trend for them to be higher in anterior than in posterior regions (anterior: M = 45.84 pmol/L, SD = 46.06 pmol/L; posterior: M = 27.56 pmol/L, SD = 37.03 pmol/L). This effect was further examined using a Wilcoxon signed-ranks test, which demonstrated that Aβ-42 levels were significantly higher in anterior regions compared with posterior regions (Z = −2.243, *p =* 0.025). There was not a significant interaction between group and region (Group x Region interaction: F(1,15) = 2.246, *p* = 0.155). Significant positive correlations were found between anterior white matter levels of Aβ-42 and neuritic plaque severity rating in the mid-frontal cortex, pre-central cortex, inferior parietal cortex, hippocampal CA1, subiculum, entorhinal cortex, transentorhinal cortex, inferior temporal cortex, the amygdala and the basal forebrain (Table 2). Posterior white matter levels of Aβ-42 were positively correlated with neuritic plaque severity rating in the mid-frontal cortex, superior temporal cortex, inferior parietal cortex, hippocampal CA1, entorhinal cortex, transentorhinal cortex, inferior temporal cortex and the amygdala (Table 2). After statistically controlling for total cortical neuritic plaque severity, average white matter Aβ-42 levels were still higher in AD patients than in non-AD controls (main effect of Group: F(1,14) = 5.184, *p* = 0.039). There was still neither a main effect of region (F(1,14) = 1.513, *p =* 0.239) nor an interaction between group and region (Group x Region interaction: F(1,14) = 3.237, *p* = 0.094) after controlling for total cortical neuritic plaque severity.

## Discussion

The current study investigated whether levels of soluble Aβ peptide are increased in the white matter of AD patients compared with non-AD controls. Levels of both soluble Aβ-40 and Aβ-42 were higher in the white matter of AD patients, even after statistically controlling for cortical neuritic plaque severity. While no regional white matter differences were found in Aβ -40, Aβ -42 levels were higher in anterior regions than in posterior regions across both subject groups.

The finding that soluble Aβ-40 and Aβ-42 levels are increased in the white matter of AD patients is consistent with the hypothesis that the white matter degeneration seen in AD could be due, in part, to the toxicity of Aβ peptides [4]. A recent study by Horiuchi and colleagues found that soluble Aβ at 10 μM or higher reduced the survival of mature oligodendrocytes and affected myelin sheet formation *in vitro* [56]. Similarly, Roher and colleagues demonstrated that increased levels of Aβ peptides are associated with reductions in multiple myelin biochemical markers and axonal demyelination [57]. In a triple transgenic mouse model, Aβ-42 led to loss of myelin integrity and increased apoptotic cell death of mouse oligodendrocyte precursor cells *in vitro*, an effect that was reversed through the use of a viral vector-conjugated Aβ-42 specific intracellular antibody [58]. Amyloid beta may have detrimental effects on white matter via oxidative stress and the activation of transcription factors, such as NF-κB and AP-1, which may cause tissue damage through a pro-inflammatory reaction [59]. Amyloid beta peptide may also activate neutral sphingomyelinase, leading to increases in the apoptogenic mediator ceramide [60].

The current study found significant correlations between Aβ42 levels and neuritic plaque severity ratings in several cortical regions. Given the amyloid cascade hypothesis and the large increases in Aβ42 known to occur in AD [2,38], this relationship is not surprising. Aβ42 is the predominant form of Aβ in parenchymal plaques [53,54] and Aβ42 displays enhanced neurotoxicity compared to Aβ40 [61-63]. Additionally, Aβ42 is known to form fibrils faster than Aβ40 [64]. While Aβ40 exists as monomers, dimers, trimers and tetramers in rapid equilibrium, Aβ42 preferentially forms pentamer and hexamer units, which assemble into higher-order oligomers and early protofibrils [65,66]. Hence, it was not unexpected that white matter Aβ42 levels would be related to the severity of cortical plaque pathology. It is noteworthy, however, that levels of Aβ-40 and Aβ-42 were increased in AD patients compared with non-AD controls, even after statistically controlling for cortical neuritic plaque severity, indicating that white matter levels of Aβ are higher in AD patients than non-AD controls, above and beyond cortical plaque pathology. These observations suggest that white matter Aβ peptides contribute to disease pathophysiology, at least in part, independent of grey matter pathology. This observation is consistent with several recent studies that suggest that, rather than being secondary to grey matter degeneration (i.e. Wallerian-like degeneration), white matter structural changes in AD might precede the emergence of grey matter pathology [4]. Several recent neuroimaging studies showed the presence of widespread white matter deficits in patients with amnestic mild cognitive impairment (aMCI) [67], subjective cognitive impairment [68] and cognitively normal individuals who go on to develop aMCI [21], despite a lack of grey matter cortical atrophy. Neuropathological studies also indicated pathological alterations in white matter preceding grey matter atrophy [23,33]. In one neuropathological study, while widespread white matter degeneration was observed in the brains of preclinical AD patients, the grey matter structure was relatively unaltered; conversely, the brains of patients with end-stage AD showed both grey and white matter changes [27]. Taken together, these findings would suggest that white matter disease might be an early event in the pathogenesis of AD. In fact, Castano and colleagues recently hypothesized that AD pathogenesis may actually begin in the white matter with oligodendrocyte dysfunction and associated pathological changes precipitating AD dementia and its progression [26]. The investigation of the pathophysiological mechanisms of white matter structural impairments could thus be critical for the identification and treatment of individuals at a high risk for developing AD.

In addition to the current findings, several other studies highlighted the importance of frontal lobe Aβ levels in disease pathology. In a postmortem study of patients with AD, Chalmers and colleagues reported that parenchymal amyloid load was more predictive of white matter degeneration in the frontal lobe than degenerative vascular disease, cerebral amyloid angiopathy or APOE ε4 genotype [69]. Similarly, postmortem studies using antibodies to Aβ identified the frontal cortex as a location for high amounts of amyloid deposition [70]. This observation is consistent with [^11^C]-PIB PET scan studies, where the highest amount of uptake has been observed in the frontal cortex, indicating more severe amyloid deposition in the grey matter in this area [71]. It is important to note, however, that PIB scans can only reliably measure fibrillar forms of amyloid in grey matter regions. In the current study, soluble Aβ levels were studied exclusively in white matter tissue. Neuroimaging studies from our group reported that it is WMH burden in posterior regions, not the frontal lobe, that is most predictive of incident clinical AD in the community and of the rate of cognitive decline in AD [9,10,35]. Thus, it is possible that, while amyloid may have a propensity to deposit in frontal regions, it is macrostructural changes in posterior areas that may drive the clinical presentation of the disease.

One potential limitation of the current study is the relatively heterogeneous control group. In future studies, it would be interesting to include a larger number of neuropathologically normal controls, in order to investigate more thoroughly how white matter pathology differs between normal, healthy aging and AD. Another potential limitation of the study is that the concentration of soluble Aβ in the cortex was not measured. While it is possible that cortical levels of soluble Aβ may be more appropriate for direct comparison with white matter levels of soluble Aβ, previous studies have suggested that there is a reliable correlation between cortical levels of soluble Aβ and both diffuse and neuritic plaques in the cortex in AD [72,73]. Hence, in the current study, we chose to use a rating of neuritic plaque severity as our marker of cortical amyloid pathology. Given the growing body of evidence that it is the levels of soluble Aβ oligomers, rather than insoluble Aβ fibrils, that correlate with the extent of synaptic loss and the severity of cognitive impairment in AD [38,39,43], however, future studies should also investigate the correlation between cortical and white matter levels of soluble Aβ oligomers.

## Conclusion

The current study found that both Aβ -42 and Aβ -40 levels were higher in the white matter of AD brains compared with non-AD controls, even after statistically controlling for cortical neuritic plaque severity. These findings suggest that Aβ peptides in white matter tissue accumulate independent of overall grey matter fibrillar amyloid pathology and are not simply a reflection of overall amyloid burden. Thus, white matter Aβ peptides may contribute to disease pathophysiology, at least in part, independent of grey matter pathology. Additionally, while no regional white matter differences were found in Aβ -40, Aβ -42 levels were higher in anterior regions than in posterior regions across both subject groups. This observation may suggest that Aβ -42 has a propensity to deposit in frontal regions, although it is not yet clear if this is related to cognitive change, or if macrostructural changes in posterior areas, likely related to cerebrovascular or other factors, may be more important for the clinical presentation of the disease.

In conclusion, the results of the current study help to shed light on a potential pathological mechanism that may contribute to white matter abnormalities in AD. Given that white matter change may be an early marker of disease, preceding grey matter atrophy, understanding the mechanisms and risk factors that may lead to white matter damage could provide a novel mechanism by which to identify those at high risk, allowing us to intervene earlier in the pathogenic process.

## Abbreviations

AD: Alzheimer’s disease
Aβ: Amyloid beta
NFT: Neurofibrillary tangle
MRI: Magnetic resonance imaging
WMH: White matter hyperintensity
DTI: Diffusion tensor imaging
ANOVA: Analysis of variance
aMCI: Amnestic mild cognitive impairment
NIA-RI: National Institute on Aging and Reagan Institute
NIA-AA: National Institute of Health-Alzheimer’s Association
